# High expression of Ras‐specific guanine nucleotide‐releasing factor 2 (RasGRF2) in lung adenocarcinoma is associated with tumor invasion and poor prognosis

**DOI:** 10.1111/pin.13069

**Published:** 2021-03-11

**Authors:** Tomoki Nakagawa, YunJung Kim, Junko Kano, Yoshihiko Murata, Zeinab Kosibaty, Masayuki Noguchi, Noriaki Sakamoto

**Affiliations:** ^1^ Department of Pathology University of Tsukuba Hospital Ibaraki Japan; ^2^ Doctoral Program in Biomedical Sciences, Graduate School of Comprehensive Human Sciences University of Tsukuba Ibaraki Japan; ^3^ Department of Diagnostic Pathology, Faculty of Medicine University of Tsukuba Ibaraki Japan; ^4^ Tsukuba Human Tissue Diagnostic Center University of Tsukuba Hospital Ibaraki Japan

**Keywords:** ECT2, lung adenocarcinoma, RasGRF2

## Abstract

The expression of Ras‐specific guanine nucleotide‐releasing factor 2 (RasGRF2) in lung adenocarcinomas was examined using immunohistochemistry in relation to clinicopathological characteristics and prognosis. In comparison to low expression, high expression of RasGRF2 was more closely associated with poor prognosis. Interestingly, expression of phosphorylated epithelial cell transforming 2 (pECT2), which – like RasGRF2 – is also a guanine‐nucleotide exchange factor, was also associated with prognosis, and patients with high expression of both RasGRF2 and pECT2 had a much poorer outcome than those who were negative for both.

**Abbreviations**
Cdc42: Cell division control protein 42
ECT2: epithelial cell transforming 2
GEF: guanine‐nucleotide exchange factor
MMP9: Matrix Metalloproteinase‐9
NMDA: *N*‐Methyl‐d‐Aspartate
pECT2: phosphorylated epithelial cell transforming 2
RasGRF1: Ras‐specific guanine nucleotide‐releasing factor 1
RasGRF2: Ras‐specific guanine nucleotide‐releasing factor 2

## INTRODUCTION

Ras family oncoproteins are activated by proteins known as guanine‐nucleotide exchange factors (GEFs). Ras‐specific guanine nucleotide‐releasing factors 1 and 2 (RasGRF1 and RasGRF2) are both mammalian Ras GEFs.^1^ RasGRF2 was originally identified by Fam *et al*. as a novel GEF for Ras.^2^ It is mapped to human chromosome 5q13 and has a multi‐domain with dual Ras GEF and Rac GEF activities.^1^ Calvo *et al*. have demonstrated that RasGRF2 also plays a role in modulating tumor cell movement and invasion by inhibiting the activation of cell division control protein 42 (Cdc42), a key component of actomyosin‐contractility‐dependent tumor cell movement.^3^

With regard to the role of RasGRF2 in carcinogenesis and cancer progression, aberrant methylation and reduced expression of RasGRF2 have recently been observed in non‐small cell lung cancers,^4^ mammary carcinomas^5^ and benign colorectal adenomas.^6^ On the other hand, Lu *et al*. have reported that RasGRF2 promotes the migration and invasion of colorectal cancer cells by modulating the expression of matrix metalloproteinase‐9 (MMP9) through the Src/Akt/NF‐kB pathway.^7^ These apparently conflicting observations may be due in part to the limited number of studies of RasGRF2.

In the present study, we examined that the expression of RasGRF2 in lung adenocarcinoma using immunohistochemistry. We also compared the expression of RasGRF2 with one of the other Ras GEFs, phosphorylated epithelial cell transforming 2 (pECT2) and showed that overexpression of both GEFs had a synergistic effect on patient outcome.

## MATERIALS AND METHODS

### Clinical samples and cell lines

Samples of 179 lung adenocarcinomas were obtained from patients who had undergone surgical resection at the University of Tsukuba Hospital (Ibaraki, Japan) between 1999 and 2007. All tissue specimens had been fixed with 10% formalin and embedded in paraffin. All cases were classified histologically according to the World Health Organization (WHO) classification (4th edition) and tumor–node–metastasis (TNM) staging was performed in accordance with the Union for International Cancer Control (UICC) system (8th edition). This research was approved by the ethics committee of University of Tsukuba Hospital.

### Immunohistochemistry

For immunohistochemistry (IHC), we used 3‐µm‐thick sections cut from formalin‐fixed, paraffin‐embedded blocks. IHC staining was performed using the Autostainer Link 48 platform (Agilent Technologies, Santa Clara, CA, USA). Deparaffinization, rehydration and target retrieval were performed in Dako PT Link using EnVision FLEX High pH Target Retrieval Solution (Agilent Technologies). The slides were incubated with a rabbit polyclonal antibody against RasGRF2 (1:100; Abcam, Cambridge, UK) and with a rabbit polyclonal antibody against ECT2 (1:200; Millipore, Billerica, MA, USA). The sections were subsequently incubated with the secondary antibody (Dako EnVision FLEX system; Agilent Technologies) and detected using DAB (Dako DAB+Liquid; Agilent Technologies).

### Evaluation of IHC

For assessment of RasGRF2 immunoreactivity, we evaluated RasGRF2 in the cytoplasm on the basis of the *H*‐score,^8^ which is defined as the summed percentage of cells at a specific intensity of staining (negative 0, weak 1+, strong 2+; Supplementary Fig. S1), calculated using the formula: [1 × (% area 1+) + 2 × (% area 2+)]. On the basis of the *H*‐score, RasGRF2 staining was classified as high staining (HS) or low staining (LS) for statistical analysis.

For assessment of pECT2 immunoreactivity, 1000 tumor cells were evaluated for the most intense cytoplasmic staining (hot spot) and evaluated as reported previously.^9^ The polyclonal anti‐ECT2 antibody reacts with both ECT2 and pECT2. Since pECT2(T790) is localized in the cytoplasm and cell membrane and not in the nucleus, we disregarded the nuclear staining of ECT2. Normal alveolar epithelial cells were used as a negative control for pECT2 staining.

### Western blot analysis

We performed Western blot analysis (WB) using six fresh surgical specimens including three HS cases and three LS cases based on the results of IHC. Protein samples extracted from the fresh tissues were separated by SDS‐PAGE.

### Statistical analysis

For all statistical analyses, SPSS 26 (SPSS, Chicago, IL, USA) was used. For RasGRF2 expression, receiver operating characteristic (ROC) curve analysis was performed and the best cut‐off points were determined at a coordinate of 100 for both disease‐free survival (DFS) and overall survival (OS). Correlations between clinicopathological features and RasGRF2 expression were analyzed by *χ* ^2^ test. The Kaplan–Meier method was used for calculation of survival curves, and the log‐rank test was performed for comparisons. Multivariate analysis was performed using the Cox proportional hazards model. Differences were considered statistically significant at *P* ≤ 0.05.

## RESULTS

### RasGRF2 expression in lung adenocarcinoma

**Figure 1:**
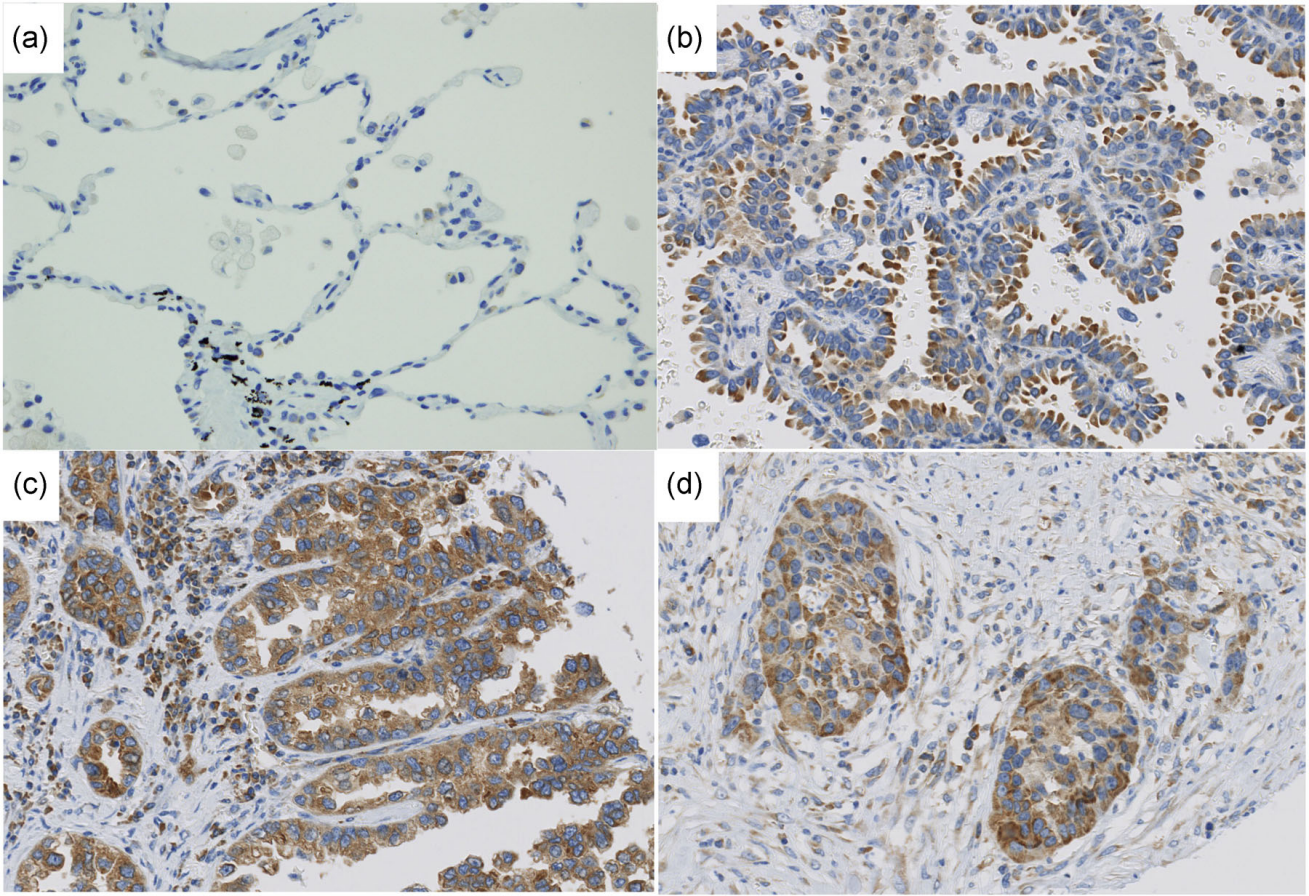
Immunohistochemistry for RasGRF2 in lung adenocarcinoma. (**a**) Normal lung parenchyma, (**b**) lepidic adenocarcinoma, (**c**) papillary and acinar adenocarcinoma, (**d**) solid adenocarcinoma.

Among the 179 lung adenocarcinomas, 100 showed high staining (HS) and the other 79 showed low staining (LS) (Fig. 1, Table 1). To confirm the IHC results for RasGRF2 expression, we performed WB using six cases (3 HS and 3 LS cases). As shown in Supplementary Fig. S2, the RasGRF2 signal was detected at about 120 kDa and the signal for HS cases was stronger than that for LS cases.

**Table 1:** RasGRF2 expression and clinicopathological features in patients with lung adenocarcinoma

		RasGRF2		
Clinicopathological features	Total patients	LS	HS	*P* value
Total patients	179	79	100	
Age (years)				0.170
≤60	44	19	25	
>60	135	43	92	
Sex				0.006
Male	103	27	76	
Female	76	35	41	
Smoking (*n* = 169)				0.010
Non‐smoker	61	28	33	
Smoker	108	29	79	
Pathological stage (Stage 0, I vs. others)				0.012
pStage 0	26	17	9	
pStage I	95	34	61	
pStage II	30	13	17	
pStage III	26	11	15	
pStage IV	2	0	2	
T factor (Tis, T1 vs. others)				0.007
Tis	25	19	6	
T1mi	10	9	1	
T1a	3	2	1	
T1b	32	19	13	
T1c	18	11	7	
T2a	60	26	34	
T2b	3	2	1	
T3	26	12	14	
T4	2	0	2	
Lymph node status				0.003
N0/N*x*	135	55	80	
N1/N2	44	7	37	
Pleural invasion				0.017
pl0	121	49	72	
pl1‐3	58	13	45	
Vascular invasion				0.008
V0	106	67	39	
V1	73	33	40	
Lymphatic permeation				0.010
Ly0	113	68	45	
Ly1	66	32	34	
Histological subtype (AIS, MIA vs. others)				<0.001
Non‐invasive adenocarcinoma				
Adenocarcinoma *in situ*	23	18	5	
Minimally invasive adenocarcinoma	10	9	1	
Invasive adenocarcinoma				
Lepidic	47	25	22	
Acinar	18	12	6	
Papillary	26	11	15	
Micropapillary	4	1	3	
Solid	30	11	19	
Invasive mucinous adenocarcinoma	21	13	8	

Abbreviations: AIS, adenocarcinoma *in situ*; HS, high staining; LS, low staining; MIA, minimally invasive adenocarcinoma.

Next, we assessed the correlation between RasGRF2 IHC and the clinicopathological features of the patients. RasGRF2 expression was found to be significantly correlated with sex, smoking, pathological stage, T factor, lymph node metastasis, pleural invasion, vascular invasion and the pathological subtype of lung adenocarcinoma (Table 1). RasGRF2 showed significantly higher expression in invasive adenocarcinomas (73/145 cases, 50%) than in non‐invasive adenocarcinomas (6/34 cases, 18%). We also examined the relationship between RasGRF2 and Kras mutation or EGFR mutation status using the TCGA database and some of the cases examined in this study (Supplementary Fig. S3 and Supplementary Table S1). The expression of RasGRF2 was not associated with either Kras mutation or EGFR mutation status.

**Figure 2:**
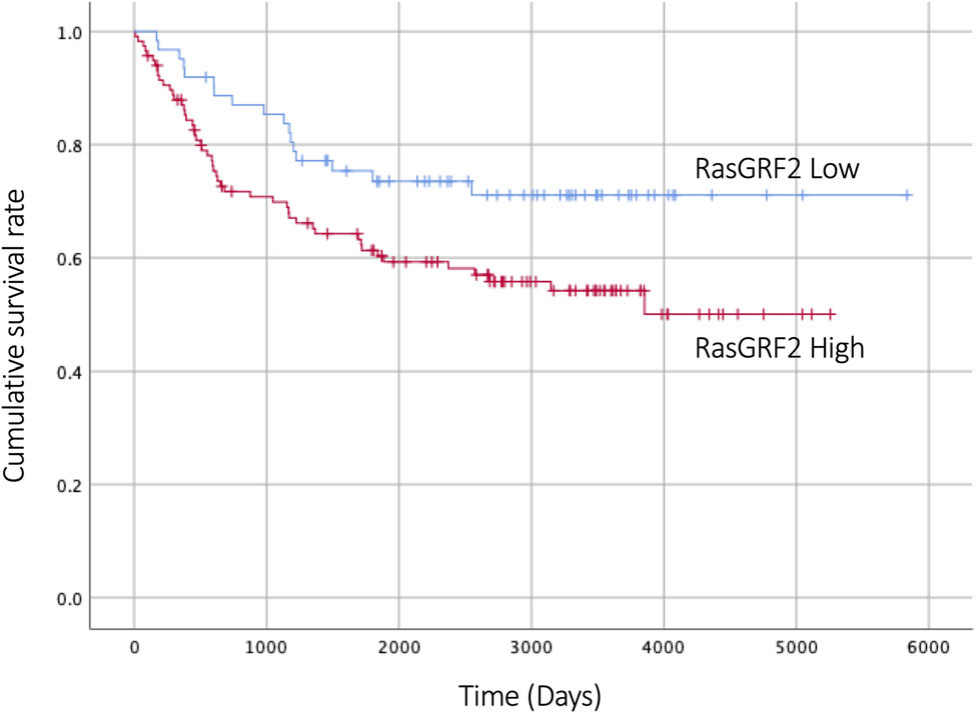
Disease‐free survival of patients with lung adenocarcinoma. Kaplan–Meier curves show that high expression RasGRF2 was significantly associated with poor outcome (*P* = 0.028).

After adjustment for age, the outcomes of LS patients and HS patients were compared. LS patients showed a more favorable outcome than HS patients (Fig. 2, *P* = 0.028). Multivariate analysis using the Cox proportional hazards model indicated that lymphatic permeation and vascular invasion were independent factors predictive of poor survival in patients with lung adenocarcinoma, whereas RasGRF2 expression was not (Table 2).

**Table 2:** Multivariate analysis using the Cox proportional hazards model

	Univariate analysis			Multivariate analysis		
Clinicopathological features	HR	95% CI	*P* value	HR	95% CI	*P* value
Age	1.014	0.585–1.759	0.960	ND		
Gender	0.406	0.234–0.705	0.001	0.879	0.477–1.621	0.679
Lymphatic permeation (0 vs. 1)	4.883	2.958–8.062	<0.001	1.815	1.019–3.235	0.043
Pleural invasion (0 vs. others)	3.931	2.419–6.387	<0.001	1.547	0.818–2.924	0.18
Vascular invasion (0 vs. 1)	7.106	4.073–12.397	<0.001	2.346	1.196–4.6	0.013
Pathological stage (I vs. others)	4.235	2.574–6.967	<0.001	1.119	0.405–3.091	0.829
T factor (T0,1 vs. others)	4.591	2.609–8.081	<0.001	1.301	0.615–2.751	0.491
*N* factor (N0/N*x* vs. N1/N2)	5.281	3.244–8.595	<0.001	1.989	0.735–5.384	0.176
Histology (AIS, MIA vs. others)	2.457	1.443–4.183	0.001	4.804	0.598–38.56	0.14
RasGRF2 (low vs. high)	1.838	1.060–3.188	0.030	0.957	0.488–1.248	0.898
Cytoplasmic ECT2 (negative vs. positive)	5.242	0.287–0.908	0.022	0.474	0.18–1.248	0.131
RasGRF2+/cytoplasmic ECT2+	0.502	0.268–0.940	0.031	1.479	0.538–4.063	0.448

Abbreviations: AIS, adenocarcinoma *in situ*; CI, confidence interval; MIA, minimally invasive adenocarcinoma; ND, not done.

### Comparison of cytoplasmic and membranous expression between pECT2 and RasGRF2

**Figure 3:**
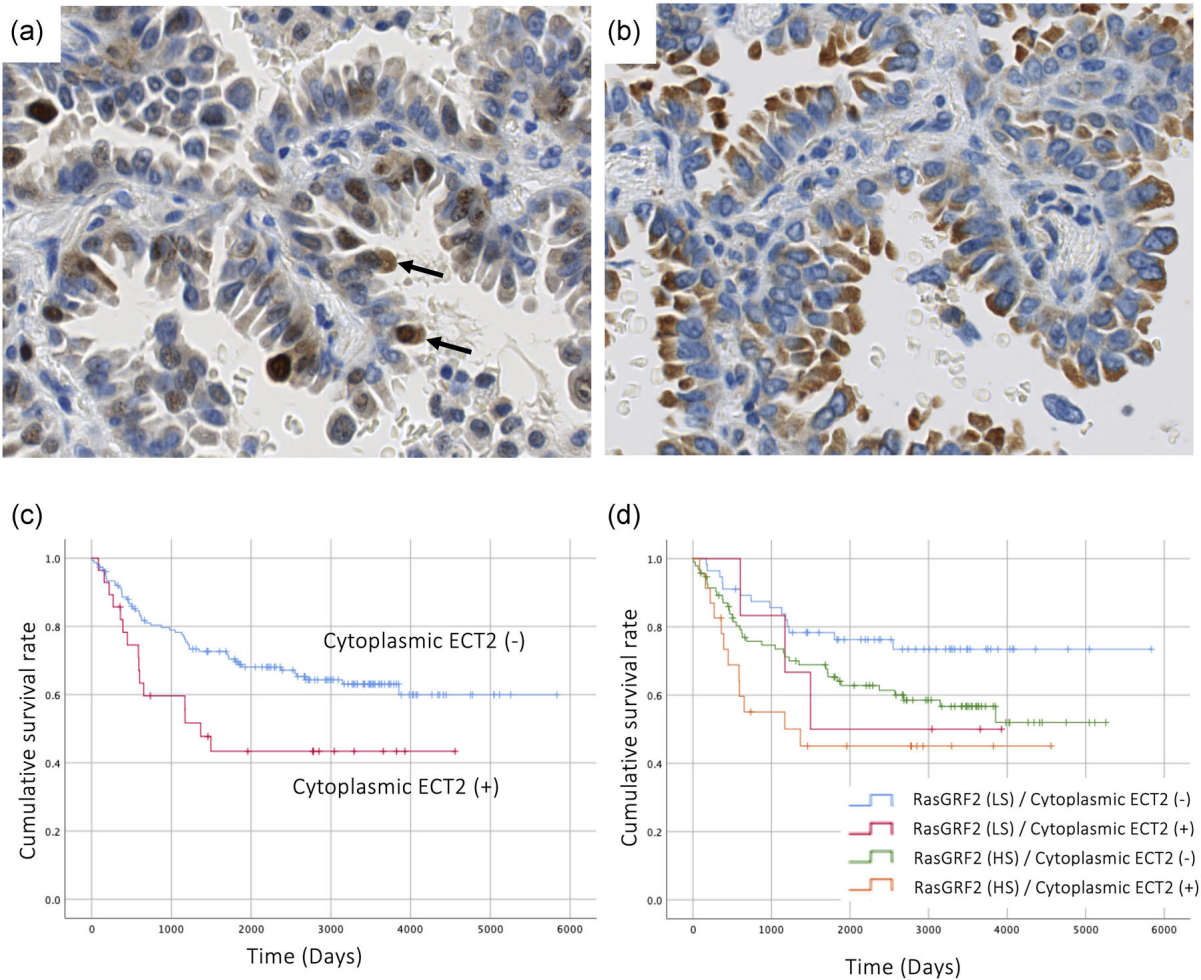
Immunohistochemistry of ECT2 (**a**) and RasGRF2 (**b**). Arrows indicated cytoplasmic ECT2 (pECT2). (**c**) Cytoplasmic ECT expression (pECT2) and patient outcome (*P* = 0.020). (**d**) Cytoplasmic expression of ECT2 and RasGRF2 and patient outcome. Cases double positive for cytoplasmic ECT2 and RasGRF2 show significantly poorer outcome than double negative cases (*P* = 0.004).

As we have reported previously, in normal cells ECT2 is localized in the nucleus and stimulates cytokinesis whereas pECT(T790) is localized in the cytoplasm and cellular membrane like RasGRF2 and functions as a Rho‐GEF. Using the same 179 cases of lung adenocarcinoma, we examined the level of pECT2 expression in the subcellular region using IHC (Fig. 3a,b). The clinicopathological characteristics of pECT2 expression are shown in Supplementary Table S2. Cases negative for cytoplasmic ECT2 (pECT2) expression showed a more favorable outcome than those that were positive (Fig. 3c, *P* = 0.020). As was the case for RasGRF2, multivariate analysis showed that pECT2 expression was not an independent prognostic factor (Table 2).

Interestingly, the expressions of RasGRF2 and pECT2 were weakly correlated and cases that were positive for both RasGRF2 and pECT2 showed a much worse outcome than cases that were negative for both (Fig. 3d, *P* = 0.004).

## DISCUSSION

In this study, we examined the expression of RasGRF2 in lung adenocarcinoma. Calvo *et al*. demonstrated that RasGRF2 plays a role in modulating tumor cell movement and invasion by inhibiting the activation of Cdc‐42.^7^ Recently, Peifen *et al*. also reported that RasGRF2 promotes migration and invasion of colorectal cancer cells.^4^ On the other hand, Chen *et al*. examined aberrant methylation of RasGRF2 in 17 human non‐small cell lung carcinomas (NSCLCs) and reported that the concordance rate between the gene expression of RasGRF2 and its aberrant methylation was 65% (11/17). They stressed that RasGRF2 expression was suppressed in lung carcinoma by aberrant methylation. In the present study, however, we found that 56% (100/179) of the lung adenocarcinomas we examined showed HS with anti‐RasGRF2 antibody. This discrepancy may have been due to the very limited number of cases examined for both expression and aberrant methylation. Furthermore, Chen *et al*. did not indicate the exact number of adenocarcinomas among the NSCLC cases they examined. Future studies will be necessary to investigate the molecular mechanisms involved in overexpression of RasGRF2 protein.

The apparent association of RasGRF2 expression with the clinical characteristics of patients with lung adenocarcinoma is interesting. Expression was higher in invasive and advanced carcinoma than in adenocarcinoma *in situ* or minimally invasive adenocarcinoma. Furthermore, high expression was associated with sex, pathological stage, T factor, lymph node status, pleural invasion and vascular invasion, and consequently correlated with poor outcome.

In this study, we also examined one of the other Rho‐related GEFs, pECT2, using the same lung adenocarcinoma cases. Both RasGRF2 and pECT2 activate Rho, thus triggering various signaling pathways associated with tumor invasion and mobility of lung adenocarcinoma. pECT2 expression was significantly associated with poor outcome of lung adenocarcinoma (Fig. 3c).

It is interesting that cases positive for both GEFs had significantly worse outcomes than cases negative for both (Fig. 3d). As RasGRF2 and pECT2 are both RhoGEFs containing the DH domain, their activation of Rho GTPase may be independent and synergistically affect cancer progression.

## DISCLOSURE STATEMENT

None declared.

## AUTHOR CONTRIBUTIONS

Conception and design of the study: TN, YK, MN, NS. Acquisition and analysis of data: TN, JK. Drafting the manuscript and figures: TN, JK, MN, NS. Administrative, technical or material support: YM, ZK.

## Supporting information

Additional Supporting Information may be found in the online version of this article at the publisher's website.

**Figure S1** Immunohistochemistry for RasGRF2 in lung adenocarcinoma. RasGRF2 was stained in the cytoplasm of non‐neoplastic bronchial epithelium (**A**) and the tumor cells (**B–D**). The *H*‐Score is defined as the summed percentage of cells at a specific intensity of staining ((**B**) negative: 0, (**C**) weak: 1+ and (**D**) strong: 2+), calculated using the formula: [1 × (% area 1+) + 2 × (% area 2+)]. RasGRF2 staining was classified as high or low expression with the *H*‐score for statistical analysis.

**Figure S2** Western blotting of RasGRF2 in lung adenocarcinomas. The target bands were probed with the anti‐human RasGRF2 primary antibody used in the IHC or with anti‐human β‐actin mouse monoclonal antibody (Sigma‐Aldrich Co.). HRP‐conjugated anti‐goat or ‐mouse IgG (Agilent Technologies) was used as the secondary antibody, respectively. All procedures were performed in accordance with the manufacturers’ instructions. Immunoreactivity was detected by chemiluminescence and captured using ChemiDoc Touch (Bio‐Rad Laboratories, Hercules, CA). Protein expression is strong in tissues of cases showing high RasGRF2 staining by IHC (high) and weak in cases showing low staining (low). Human glioblastoma U251MG cells and pure water were used as positive (PC) and negative (NC) controls, respectively.

**Figure S3** The relationship between RasGRF2 expression and Kras mutation in lung adenocarcinoma was analyzed using TCGA database. There was no significant relationship between them.

**Table S1** Relationship between RasGRF2 expression and EGFR mutation in patients with lung adenocarcinoma.

**Table S2** Cytoplasmic ECT2 (pECT2) expression and clinicopathological features in patients with lung adenocarcinoma.
